# Student-led curricular approaches in medical education: the educational effects of a virtual fundamentals of COVID-19 course

**DOI:** 10.1186/s12909-021-03076-x

**Published:** 2022-03-08

**Authors:** Megan Z. Chiu, Rolando G. Gerena, Rebekah L. Roll, Joseph M. Baker, Maritza Gomez, Cameron M. Brown, Abigail M. Brenner, Christina C. Huang, Paul Y. Ko, Margaret E. Bauer, Daniel J. Trujillo

**Affiliations:** Indiana University School of Medicine, Indianapolis, USA

**Keywords:** Curriculum, Medical Students, Self-assessment, COVID-19 Pandemic, Undergraduate Medical Education

## Abstract

**Background:**

As the field of education was adapting to virtual learning during the COVID-19 pandemic, a need quickly emerged for a course to prepare medical students for future clinical practice. This call to action was answered by creating an innovative Fundamentals of COVID-19 course at the Indiana University School of Medicine (IUSM). As a group of medical student leaders at IUSM, we developed this online course in order to support our fellow students and the community.

**Methods:**

The study examined the educational effects of completing the Fundamentals of COVID-19 course. In order to examine these effects, the study asked enrolled students to complete both a pre- and post-course self-assessment survey. Students were asked an identical set of questions on each survey about their knowledge, skills, and abilities (KSA) regarding COVID-19. Composite scores were created for each KSA learning domain. Responses were provided using a five-point Likert scale ranging from 1 = *strongly disagree* to 5 = *strongly agree*.

**Results:**

Out of the 724 students enrolled, 645 students completed both the pre- and post-course assessment surveys. Findings show that there were both meaningful and statistically significant differences in students’ responses to the pre- and post-course surveys. Results show 1.) a significant mean increase in the knowledge composite score of 1.01, 95% CI [0.95, 1.06], *t*(644) = 36.4, *p* < .001, *d* = 1.43; 2.) a significant mean increase in the skills composite score of .55, 95% CI [0.50, 0.60], *t*(644) = 20.70, *p* < .001, *d* = 0.81. and 3.) a significant mean increase of the abilities composite score of 1.02, 95% CI [.97, 1.07], *t*(644) = 36.56, *p* < .001, *d* = 1.44.

**Conclusions:**

These findings demonstrate that the student-developed, online Fundamentals of COVID-19 course resulted in notable and statistically significant educational effects. The increase in students’ self-reported ratings, especially in the knowledge and abilities domains, indicate that meaningful learning occurred within the course. These findings have notable implications for medical student training during healthcare emergencies, such as a pandemic, as well as within modern clerkship environments. Overall, our findings provide evidence that student-led curricular design and virtual delivery of course content can be effective tools in undergraduate medical education.

**Supplementary Information:**

The online version contains supplementary material available at 10.1186/s12909-021-03076-x.

## Introduction

In the spring of 2020, students at the Indiana University School of Medicine (IUSM) responded to an immediate need for new curriculum related to COVID-19. During this time, undergraduate medical students’ direct patient care responsibilities were suspended due to the pandemic. In a Letter to the Editor, “Answering the Call to Action: COVID-19 Curriculum Design by Students for Students,” we described how we, as a small group of student leaders, developed a course to prepare students for clinical clerkship experiences with COVID-19 [1]. We accomplished this goal by completing a student-led curriculum design class, Leadership in Medical Education Elective During the COVID-19 Pandemic, which was the first of its kind at IUSM. Through this class, we developed a virtual Fundamentals of COVID-19 course that all third- and fourth-year IUSM students were required to enroll in during May of 2020. With the help of IUSM faculty and educational experts, we conducted a quasi-experimental research study to evaluate the student learning that occurred from completing this course.

### Medical education during COVID-19

The COVID-19 pandemic continues to rapidly change medical students’ classroom learning and clinical education. As Hall et al. [2] pointed out, “The COVID-19 global pandemic is challenging healthcare systems in unprecedented ways, affecting not only the delivery of care, but also our delivery of medical education.” Scholarship describes that solutions to these challenges are necessary during this critical time [2–5]. Rose [4] puts forth, “While in the midst of this COVID-19 crisis, it is crucial that the academic educational community learns from the experiences and prioritizes a forward thinking and scholarly approach…” Our study directly responds to these calls for action by examining the educational effects of a virtual Fundamentals of COVID-19 course. This innovative learning intervention was led by students and presents an effective way to respond to the challenges of the COVID-19 pandemic on medical student education.

### Course design

The medical curriculum at the Indiana University School of Medicine includes 18 months of pre-clerkship foundational course work followed by clinical studies in the third and fourth years. The Fundamentals of COVID-19 course, taught while clinical studies were suspended, was intentionally designed to teach 3^rd^- and 4^th^-year medical students the knowledge, skills, and abilities (KSA), necessary to learn within and respond to the pandemic as they prepared to start or return to clerkships. The two-week virtual course was delivered via Zoom Cloud Meetings (Zoom Video Communications, San Jose, Calif.), Top Hat (Top Hat LTD, Toronto, Canada), and Canvas LSM (Instructure Inc., Salt Lake City, Utah). Asynchronous sessions included videos pre-recorded by faculty content experts, reading assignments, and participation in class discussion boards. Synchronous activities included interactive panel discussions with public health experts and with physicians focusing on evidence-based medicine, a “Grand Rounds”-style case study of a patient hospitalized with COVID-19, and a variety of interactive small group activities. The distribution of content delivery was 15% synchronous, 67% individual asynchronous, and 18% small group.

The Fundamentals of COVID-19 course adopted the backwards design approach recommended by Horst and Pendegrast [6] and focused first on developing course learning objectives (CLOs). IUSM faculty, educational experts, and student leaders developed a total of 20 specific CLOs that were mapped to our Institutional Competencies (Additional File 1) and were evaluated through knowledge (7 questions), skills (5 questions), and abilities (5 questions) domains. For example, major goals of the COVID-19 course included students becoming knowledgeable about the virology and immunology of COVID-19, and students gaining the ability to identify at-risk populations for COVID-19. Overall, the student-led curriculum design, virtual implementation, and the overall scale made the Fundamentals of COVID-19 course at IUSM a compelling educational intervention for academic research.

### Student-led curricular development

The argument for student-led curriculum has been discussed numerous times over the years, but there has been limited implementation of this curricular approach at medical institutions, including IUSM [7], until very recently [8–10]. At a basic level, a student-led curriculum provides the tools to help medical students become better teachers [11]. Research suggests that exposure to teaching principles, techniques, and skills should begin in medical school and continue through residency and clinical practice [12]. Furthermore, recent literature highlights the advantages of the student perspective as students have a “heightened sense” of curricular gaps and thus are able to “capture different sets of needs…via a student-centered approach [9].” Students at the Johns Hopkins University School of Medicine have a Student Curriculum Review Team (SCRT) that provides a pathway for student-feedback to play an integral role in curriculum development [13]. The SCRT contends that, “As consumers of education, students have the right and responsibility to be involved in curricular reform and communicate their ideas freely [13].” Taken together, these perspectives demonstrate the benefits of student-led curriculum and its increasing importance to health sciences education.

### Virtual delivery of course content

The IUSM Fundamentals of COVID-19 course was largely shaped by the need to deliver new curriculum in a completely virtual format. Online curriculum has become increasingly popular due to increased demands for faculty productivity, resulting in decreased time for traditional teaching methods, especially in the face of a pandemic [14]. Educators are now more than ever leveraging the use of technology in undergraduate and graduate medical education [15]. For example, Anupan et al. describe the use of rapid design thinking as a way to overcome COVID-19 challenges in medical education [3]. Within this framework, “the goal is to develop accelerated solutions that are also human-centered or enhance the user experience”; and leveraging digital technology is described as the primary way to achieve these goals [3]. This progressive framework also helped guide the student-led development of the Fundamentals of COVID-19 course.

### Study goals

The goal of the current study was to examine the educational effects of the Fundamentals of COVID-19 course at IUSM. This course was unique because it was developed by students to teach essential principles of the COVID-19 pandemic in an online format. Specifically, the aim of the study was to determine if students enrolled in the course learned key knowledge, skills, and abilities (KSA) related to COVID-19. We were also interested in student satisfaction with the virtual delivery of class content and the course overall. The research questions guiding this study were: 1) Did students increase their KSA related to COVID-19 as a result of completing the Fundamentals of COVID-19 course at IUSM? and 2) Were students generally satisfied with the Fundamentals of COVID-19 course? We hypothesized that the students would report moderate student learning gains in the KSA related to COVID-19 on the post-course assessment survey compared to the pre-course assessment survey and that the students would be moderately satisfied with the course.

## Methods

### Research framework

The Fundamentals of COVID-19 course at IUSM was implemented in May 2020 as a required course for all 724 third- and fourth-year medical students. Subsequently, a quasi-experimental research design was implemented to understand student learning in this course. All 724 students enrolled in the course were eligible to participate in the study by completing both a pre-course and post-course assessment survey. Those students who declined to participate or who only completed one of the two surveys were excluded from the study. This type of research design, characterized by the use of pre- and post-course surveys, has been used effectively in medical education and general higher education to assess the success of new learning interventions [16, 17]. Additionally, Pohlmann and Boggs’s foundational work on the validity of self-reported measures of academic growth support this type of research framework [18]. The surveys were developed to measure students’ KSA, as they relate to COVID-19. The two surveys were directly connected to the CLOs and contained an identical set of KSA self-assessment question items. We examined the matched dataset of student ratings to identify statistically significant differences in students’ self-assessment ratings prior to and after completing the course. The self-assessments intentionally asked the students to rate their confidence with completing specific skills and abilities because the students were unable to practice their clinical skills in an in-person teaching environment due to the pandemic.

### Questions and likert scale

The students completed the two assessments by rating their level of agreement with 17 statements that measured their KSAs related to COVID-19, as shown in Additional File 2. Although not validated, due to time constraints, the survey was developed by a team of experts at IUSM and was guided by the assessment principles for higher education put forth by Banta, Jones, & Black as well as Zahl, Jimenez, and Huffman’s considerations for assessments in professional degree programs [19, 20]. Students also responded to additional questions on the post course survey which asked about topics that could only be evaluated by the students after they completed the course, such as the overall success of the course and the effectiveness of the virtual delivery of course content. The students rated their level of agreement with all the question items using a five-point Likert scale: 1 = *Strongly Disagree*; 2 = *Disagree*; 3 = *Neutral*; 4 = *Agree*; 5 = *Strongly Agree*.

### Ordinal and interval data and composite scores

The ordinal data collected were converted to numbers (e.g., 3 = Neutral) and treated as interval data for the purpose of statistical analysis. Composite scores were created by averaging the student ratings across all questions of a specific learning domain. Non-weighted averages were calculated for both the pre- and post- course surveys. This resulted in both a pre- and post- composite score for each KSA learning domain. Kane and Case explain that the individual components of a composite score should not be weighted unless there is a clear reason and that it would increase reliability and validity [21]. At a basic level, composite scores are often used in education and can be seen in, for example, grade point averages (GPA). The use of composite variables in the assessment of health professions programs is also common. We followed Song, Lin, Ward, and Fine’s instructions for using them within this educational context [22]. Overall, the use of composite scores provided an effective way to consider the change in students’ self-assessment ratings over time.

### Statistical analysis

Paired-samples t-tests were used to determine if there were statistically significant mean differences between students’ self-assessment scores on the pre-course survey compared to the post-course survey for each learning domain. The within-subject parametric tests compared the two matched groups of the independent variable (IV), pre- and post-course survey responses, on one dependent variable (DV). The DV changed for each test to include a specific composite score – knowledge, skills, or abilities. Paired-samples t-tests were also conducted on individual question items in order to learn more about student learning of COVID-19.

The paired-samples t-tests met the required statistical assumptions. For each test, there were no significant outliers in the differences between the matched pre- and post- course survey groups. Additionally, for each test, the assumption of normality was not violated, as assessed by visual inspection of Normal Q-Q plots. Finally, a Bonferroni correction was made on the preset probability (*p*) values of the paired-samples t-tests [23, 24]. The correction was made because conducting multiple statistical tests exponentially increases the risk of Type 1 error. The preset probability value (*p*) of the statistical tests in this study was 0.05. After the Bonferroni correction was applied (0.05 / 17 question specific t-tests), the preset *p* value was adjusted to 0.003 and then used throughout the study. All statistical analyses were conducted using Statistical Product and Service Solutions (SPSS Statistics, International Business Machines Corporation, Armonk, New York) software, version 26.

### Ethical considerations

Approval for this study was obtained from the Indiana University, Institutional Review Board (IRB). The protocol number is 2004320338. This approval ensured ethical safeguards and confirmed that the study procedures were carried out under all appropriate guidelines, regulations, and policies. The study participants were informed that their survey responses would be used for both research and institutional improvement purposes. Informed consent was obtained from all study participants. The IRB approval contained exempt status for the study so that participants’ confidentiality would not be placed at risk by collecting written documentation. The study was implemented within a required course. Students were informed that their decision to not complete the survey would not affect their grades or academic standing. Students also had the opportunity to complete the survey but for their answers to not be used for research purposes. Forty-six students chose not to complete the pre-course survey, 43 students chose not to complete the post course survey, and no students chose to take the survey but for their data to not be used for research purposes. The study adhered to all ethical standards of human subjects research and was performed in accordance with the Declaration of Helsinki.

## Results

### Respondent characteristics

Of the 724 students enrolled in the course, 678 (93.6%) completed the pre-course survey, and 681 (94.1%) students completed the post-course survey. The matched dataset used in analysis only included the 645 (89.1%) students who completed the self-assessment on both the pre- and post- course surveys. The matched data set includes 48.4% (312) third-year and 51.6% (333) fourth-year medical students. Demographics of students in the dataset are similar to the demographic characteristics of IUSM students overall: 45.9% (296) of respondents self-identified as female, 51% (329) as male, 0.3% (2) as nonbinary, and 2.8% (18) indicated that they prefer not to answer questions about gender identity. Additionally, 25.3% (163) respondents self-identified as minority students, and 67.1% (433) as white students. Lastly, 7.6% (49) of students chose not to answer demographic questions about race and ethnicity.

### Findings

**Fig. 1:**
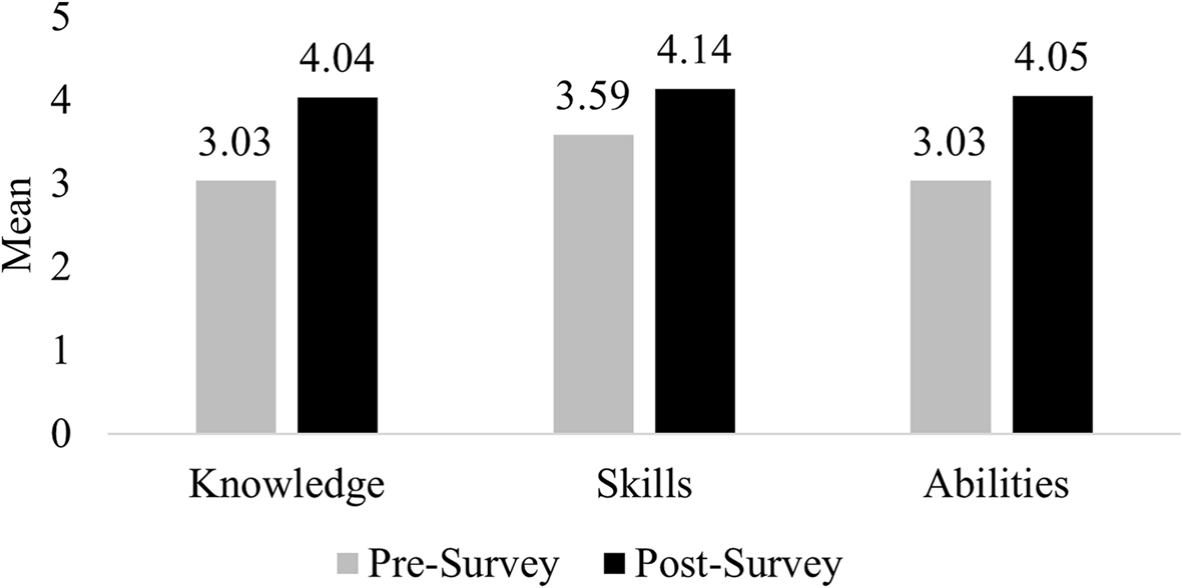
Composite score means of student self-reported knowledge, skills, and abilities before and after completing the Fundamentals of COVID-19 course. The mean pre-course (gray) and post-course (black) survey results are shown for each learning domain. Within each learning domain, the post-course survey increase was statistically significant (*p* < .001)

Overall, results show higher student ratings on the post-course survey compared to the pre-course survey across all KSA learning domains. For example, composite score means for each KSA learning domain show an increase in students’ self-assessment ratings after the course was completed compared to the matched pre-course survey results (Fig. 1). These results are both statistically significant (*p* < 0.001) and substantial as measured by Cohen’s *d*. Subsequently, details associated with these results are presented. Students’ assessment of learning gains in knowledge associated with COVID-19 are presented first. This is followed by findings associated with students’ assessment of learning gains in skills and abilities associated with COVID-19.

### Knowledge

**Fig. 2:**
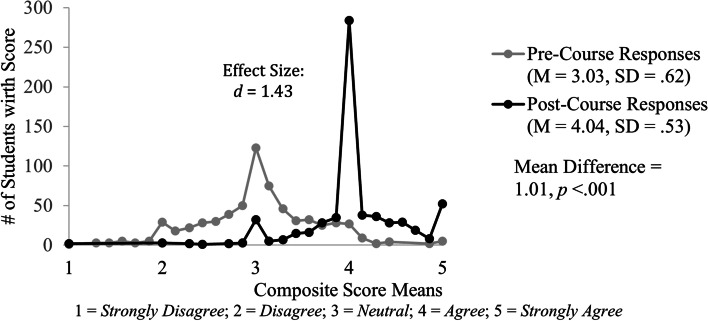
Distribution of composite score means for students’ self-reported knowledge of COVID-19 before and after completing the new- student-led course. The distribution is shown for pre-course (gray) and post-course (black) survey results. The post-course survey aggregate mean increase of 1.01 was both substantial (*d* = 1.43) and statistically significant (*p* < .001)

Results from a paired-samples t-test show students responded with higher self-assessment ratings about their knowledge of COVID-19 on the post-course survey (M = 4.04, SD = 0.53) compared to the pre-course survey (M = 3.03, SD = 0.62) with a significant mean increase of the knowledge composite score of 1.01, 95% CI [0.95, 1.06], *t*(644) = 36.44, *p* < 0.001, *d* = 1.43 (Fig. 2).

It is notable that the effect size, as measured by Cohen’s *d*, is large (1.43) indicating that students’ scores on the pre- and post-course self-assessments are substantially different. Individual paired-samples t-test conducted on matched responses for the seven knowledge question items show statistically significant mean increases ranging from 0.65 to 1.36 as well as notable effect sizes (Table 1).

**Table 1:** Students pre- and post-survey responses on COVID-19 knowledge questions

	COVID-19 Knowledge	Pre-Survey (*N* = 645)		Post-Survey (*N* = 645)		Mean Difference	Effect Size
	Students' Self-Assessment Ratings	M	SD	M	SD	( ±)	*d*
K1	I am knowledgeable about virology and immunology as they relate to COVID-19	3.08	0.81	4.02	0.61	0.94***	1.08
K2	I am knowledgeable about the pathophysiology of COVID-19	2.88	0.82	3.98	0.63	1.10***	1.20
K3	I am knowledgeable about the impact of population health in the context of a pandemic, and in particular for COVID-19	3.39	0.83	4.18	0.63	0.79***	.90
K4	I am knowledgeable about the impact of social determinants of health in the context of a pandemic, and in particular for COVID-19	3.57	0.87	4.22	0.63	0.65***	.71
K5	I am knowledgeable about the ways individuals and organizations can advocate at the state and national level during epidemics/pandemics	2.81	0.90	3.96	0.66	1.15***	1.13
K6	I am knowledgeable about the legal aspects that impact COVID-19 patients, providers, and the community during the pandemic	2.47	0.87	3.83	0.74	1.36***	1.33
K7	I am knowledgeable about the issues surrounding utilization and preservation of finite resources that impact patients, providers, and the community during the pandemic	3.03	0.87	4.07	0.63	1.04***	1.07
*Knowledge Composite Rating Score*		3.03	0.62	4.04	0.53	1.01***	1.43

^***^ *p* < .001

### Skills

**Fig. 3:**
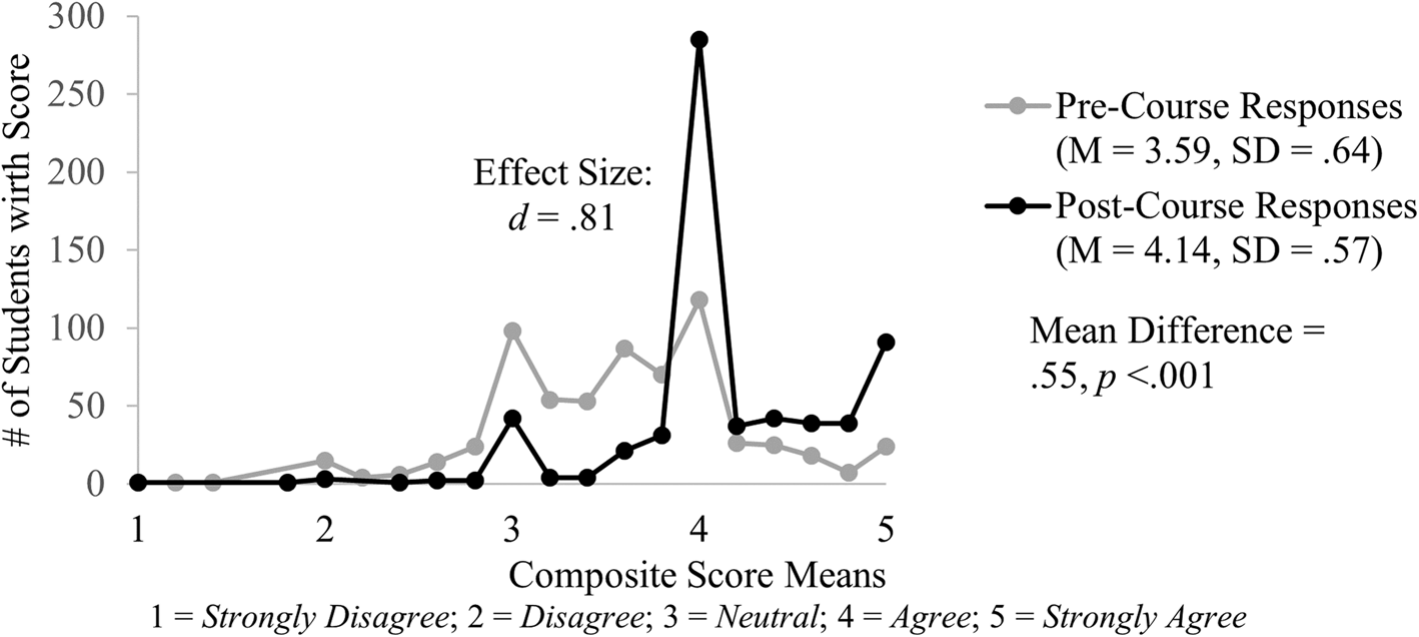
Distribution of composite score means for students’ self-reported skills related to COVID-19 before and after completing the student-led course. The distribution is shown for pre-course (gray) and post-course (black) survey results. The post-course survey aggregate mean increase of .55 was notable (*d* = .81) and statistically significant (*p* < .001)

Students responded with higher self-assessment ratings about their skills related to COVID-19 on the post-course survey (M = 4.14, SD = 0.57) compared to the pre-course survey (M = 3.59, SD = 0.64). This is a significant mean increase of the skills composite score of 0.55, 95% CI [0.50, 0.60], *t*(644) = 20.70 *p* < 0.001, *d* = 0.81 (Fig. 3).

The effect size, as measured by Cohen’s *d* is moderate to large showing that students’ scores on the pre- and post-course surveys are notably different. Results from individual paired-samples t-test conducted on matched responses for the five skills question items also show statistically significant differences as well as moderate to large effect sizes (Table 2). Findings related to the skills associated with COVID-19, as well as the other learning domains, are reviewed further in the discussion section.

**Table 2:** Students pre- and post-survey responses on COVID-19 skills questions

	COVID-19 Skills	Pre- Survey (*N* = 645)		Post-Survey (*N* = 645)		Mean Difference	Effect Size
	Students' Self-Assessment Ratings	M	SD	M	SD	( ±)	*d*
S1	I am confident in my skills to use principles of evidence-based medicine, including biostatistics, to evaluate efficacy of therapeutic interventions for COVID-19 infection	3.37	0.94	4.05	0.69	0.68***	.72
S2	I am confident in my skills to analyze the management of epidemics and pandemics historically and in modern medicine	3.14	0.90	4.09	0.67	0.95***	.96
S3	I am confident in my skills to identify a research question	3.98	0.77	4.26	0.67	0.28***	.36
S4	I am confident in my skills to appraise the quality and credibility of a source and synthesize the information to advance my understanding of pandemic responses	3.84	0.77	4.14	0.66	0.30***	.36
S5	I am confident in my skills to implement basic strategies for mental health and wellbeing promotion for providers in the face of a healthcare emergency, and understand their importance to overall health	3.60	0.81	4.15	0.65	0.55***	.61
	*Skills Composite Rating Score*	3.59	0.64	4.14	0.57	0.55***	.81

^***^ *p* < .001

### Abilities

**Fig. 4:**
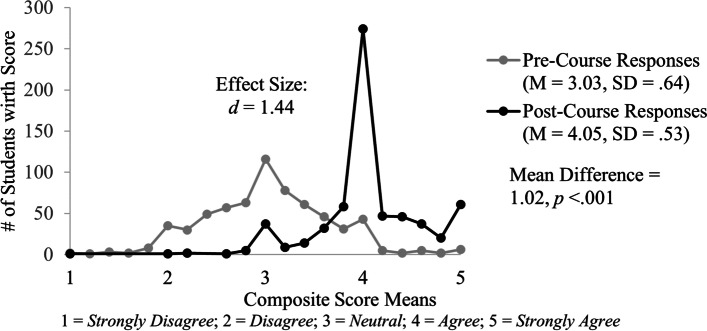
Distribution of composite score means for students’ self-reported abilities related to COVID-19 before and after completing the student-led course. The distribution is shown for pre-course (gray) and post-course (black) survey results. The post-course survey aggregate mean increase of 1.02 was both substantial (*d* = 1.44) and statistically significant (*p* < .001)

Students responded with higher self-assessment ratings about their abilities related to COVID-19 on the post-course survey (M = 4.05, SD = 0.53) compared to the pre-course survey (M = 3.03, SD = 0.64). This is a significant mean increase of the abilities composite score of 1.02, 95% CI [0.97, 1.07], *t*(644) = 36.56, *p* < 0.001, *d* = 1.44 (Fig. 4).

Similar to the findings associated with the other learning domains, the large effect size also indicates that the differences in students’ responses are substantial. Results from individual paired-samples t-test conducted on matched responses for the five abilities question items also show statistically significant differences and large effect sizes (Table 3).

**Table 3:** Students pre- and post-survey responses on COVID-19 abilities questions

	COVID-19 Abilities	Pre-Survey (*N* = 645)		Post- Survey (*N* = 645)		Mean Difference	Effect Size
	Students' Self-Assessment Ratings	M	SD	M	SD	( ±)	*d*
A1	I am confident in my ability to recognize the clinical presentation of a patient with COVID-19	3.29	0.85	4.12	0.63	0.83***	.92
A2	I am confident in my ability to outline a treatment course for suspected COVID-19 patients	2.52	0.89	3.82	0.75	1.30***	1.32
A3	I am confident in my ability to identify at-risk populations for poor outcomes with COVID-19	3.54	0.83	4.26	0.60	0.72***	.82
A4	I am confident in my ability to describe disaster medicine principles, including the processes and policies by which community and international agencies interact to coordinate a safe and effective disaster/pandemic response	2.55	0.88	3.94	0.65	1.39***	1.42
A5	I am confident in my ability to identify ways of modifying communication strategies based on the context	3.26	0.88	4.11	0.62	0.85***	.89
	*Abilities Composite Rating Score*	3.03	0.64	4.05	0.53	1.02***	1.44

^***^ *p* < .001

### Additional findings

#### Student satisfaction

The students in the sample also provided useful feedback about their satisfaction with the course and the virtual delivery of course content using the same five-point Likert scale that was used to assess the KSA learning domains. Results from the post-course survey regarding virtual delivery showed mean ratings of 4.02 (SD = 0.75) and 3.83 (SD = 0.93) for the following statements, respectively: “The technology generally worked well for this virtual course” and “The content was delivered effectively in a virtual format.” Results from the student surveys regarding overall course effectiveness showed mean ratings of 3.70 (SD = 0.996) and 3.69 (SD = 1.02) for the following statements, respectively: “Overall, this course provided an effective learning experience.” and “The virtual delivery of course content did not detract from the overall effectiveness of this class.” Taken together, these results demonstrate that students were generally satisfied with the Fundamentals of COVID-19 course and that they viewed the technology and virtual format positively. This provides a case study in support of future research about student-led curriculum design and online instruction in medical student education. Although students’ perceptions of learning gains and satisfaction cannot be fully attributed to student-led curriculum development and online instruction, they do point to the efficacy of these increasingly popular teaching methods. Overall, these findings may be most useful in that they effectively illustrate the magnitude of students’ perceived learning within these approaches and also provide a compelling example of a course embedded assessment.

## Discussion

The virtual Fundamentals of COVID-19 course resulted in notable educational effects. The purpose of the course was not just to teach students about COVID-19, but rather to equip students with the KSA to adapt to ever-changing data and to critically think about the multidisciplinary coordination needed to manage a global pandemic. Findings from our study show that the student-led Fundamentals of COVID-19 course was successful in achieving this goal. Achieving this goal was especially important as many of the medical students who completed the course are now delivering vaccines that are vital to stopping the pandemic. The analyses show statistically significant self-reported learning improvement in students’ knowledge, skills, and abilities, measured by large effect sizes in both composite scores and individual questions. The results also show that the students who completed the virtual Fundamentals of COVID-19 course were satisfied with the course and its virtual delivery. Student improvement in confidence, especially in the knowledge and abilities domains, demonstrate that meaningful student learning occurred as a result of the course. Overall, this study’s findings imply that student-led curricular design and virtual delivery of curriculum can be effective in an undergraduate medical education setting.

### Efficacy of student-led curriculum development

Student-led curricular development offers different perspectives and insight into course design. Although this study does not provide comparative data between student-led vs faculty-led curricular development, the overall student satisfaction and reported increase in learning outcomes provides a compelling institutional example of the success of student-led curricular design. This example aligns with current medical education research regarding the benefits of student involvement in curricular design [9, 11, 14]. Additionally, the benefits of student-led curricular design are parallel to those of peer teaching, which has been shown to have a “positive impact on both the peer teacher and the learners [25].” This concept is well supported and described in medical education literature as cognitive congruence. For example, Lockespier et al. show that the ability of second year students to anticipate problems that first year students might have in understanding particular concepts was important in creating cognitive congruence [26]. Because peer teachers recently learned the material themselves, they are able to share their own struggles, learning experiences, and ultimately describe the approaches that they used to overcome those challenges [26–28]. As discussed by McCutcheon et al., “student participation” in curricular development “render[s] content more accessible to learners [25].” We consciously applied these peer teaching and learning principles as we led the student development of the Fundamentals of COVID-19 course at IUSM. Although the positive changes in student ratings from the pre- to post-course assessments are substantial and statistically significant, we cannot fully attribute a causal link between these changes and the effectiveness of the course. However, the high student satisfaction and large change in self-perceived learning do provide evidence to support the benefits of student-led curricular design.

### Efficacy of virtual delivery of course content

As discussed earlier, education, in particular medical education, will forever be different due to COVID-19. Virtual or online education has not only become a necessary aspect of learning but in many cases has become a preferred choice by educators as it saves critical resources [14] and helps ensure student health [26]. The results from our study show students tended to agree with the statement that the virtual delivery of course content was effective in the Fundamentals of COVID-19 course. Consistent with current literature on the use of educational technology, our data provide evidence to support virtual delivery as an alternative educational platform [29] and an effective tool for large audiences.

The Fundamentals of COVID-19 course clearly demonstrates that intentionally developed CLOs can be achieved through the use of a virtual delivery platform in medical education. It is important to consider that students self-reported higher ratings for question items in the skills domain on both the pre- and post-surveys (Figs. 1 and 3). These higher ratings likely reflect that skill development is an integral component of the IUSM curriculum overall. However, the relatively small increase in skill ratings between the pre- and post- course surveys may speak to the limitations of the virtual delivery platforms. These online platforms may present a barrier to skill development versus in-person learning. This consideration is contrary to results of a systematic review in nursing education examining effectiveness of traditional vs. online learning for clinical skills training, which “suggests that online learning for teaching clinical skills is no less effective than traditional means [25].” Further research is required to clarify the relationship between virtual learning and clinical skills training. Although there may be some new barriers with virtual learning, students in this course self-reported significant academic improvement across all KSA domains and also reported that this course was delivered effectively in a virtual format. These findings provide evidence in support of current literature that argues the use of online platforms to teach and disseminate medical knowledge, skills, and abilities, is effective [15]. Our study uniquely demonstrates that the use of online educational platforms can be implemented effectively on a large-scale.

### Future research

Overall, the findings from our study demonstrate that the virtual Fundamentals of COVID-19 course achieved its CLOs. Previous scholarship in the medical education literature supports the findings from our study and informs future research about student-led curriculum development and online instruction. For example, the SCRT at The Johns Hopkins School of Medicine and the Canadian Federation of Medical Students’ (CFMS) task force on homelessness are both recent examples of student-led curriculum development. Similar to our study, Hashmi et al. and Hish et al. conclude that these types of initiatives are positive experiences for students and lead to improved learning [8, 13]. It is interesting that the CFMS task force conducted an extensive literature review that examined 81 sources about evidence-based guidelines and student programs to build a new educational framework about homelessness [8]. It is also notable that the SCRT reviewed course evaluation data and surveyed faculty course directors in order to better understand curricular change at their medical school [13]. Finally, a study connected to the Medical Library Association used results from both a graded assignment and a survey to determine that students preferred and retained information from online PubMed training compared to an in-person approach [29]. Although results from these prior studies are similar to the findings and conclusions associated with our study, they use notably different types of data and approaches to research. The proactive use of an extensive literature review, surveying faculty, and using a graded assignment to assess student learning, stand out as being especially helpful to future studies. Future research should consider using these additional approaches and information to further examine how student-led curriculum development and the virtual delivery of course content contribute to positive educational outcomes.

### Limitations of study design, data collection, and analysis

It is important to consider the inherent limitations associated with this study. First, there is an innate risk of studies that use a pre- and post- survey design to collect feedback from participants. There is speculation that this research framework biases improvement as survey takers will have a social desirability bias and purposefully change their answers to positive. Lockspeiser et al. contend that students have been shown to value learning from peers, as was done in the Fundamentals of COVID-19 course, and that they actually may feel more comfortable in providing more honest appraisals of peer-developed resources [26]. However, this assertion is limited in scope and it is not commonly put forth in educational research as a factor in mitigating the effects of social desirability bias. Gonyea and Miller provide suggestions for using students’ self-reported data in higher education research and put forth that this type of data should be used to measure students’ general perceptions of learning through, for example, self-assessments or general satisfaction surveys [30]. They make clear that students’ self-reported data should not be used to measure cognitive gains. This position is strongly supported in the scholarship about measuring student learning outcomes and making value-added claims based on educational research [31, 32]. Our study was designed to collect students’ self-reported perceptions of learning because we determined that this type of data was the most accessible given the COVID-19 pandemic. Although measuring students’ perceptions of learning is not as rigorous as measuring students’ cognitive gains, it can still lead to a greater understanding of innovative curricular approaches. Gathering more direct evidence of student learning through, for example, objective structured clinical exams (OSCEs), performance-based assessments (PBAs), or other in-person skill-based examinations, will help future studies to more objectively measure educational outcomes.

This study also has additional limitations associated with its research design, data collection, and analysis procedures. For example, this study examined the results of a student self-assessment at one institution. Additionally, because all third- and fourth-year medical students were enrolled in the Fundamentals of COVID-19 course there was not a control group. As a result, it was difficult to fully identify value-added educational effects or make causal claims about the efficacy of the Fundamentals of COVID-19 course at IUSM. This lack of a control group also prevents direct comparison of student-led versus faculty-led curriculum designs. Subsequent research should prioritize using control groups so that these types of direct comparisons can be made, and the benefits of student-led curriculum and online platforms can be better understood. Once again, a limitation of this study is that we asked students to self-assess their learning as part of the pre- and post-course assessment surveys. As a result, we measured student outcomes according to perceptions and not through more objective measures. Although this information is valuable, future research should include more direct evidence of student learning and seek to triangulate findings from multiple data sources.

Additionally, students’ survey responses were examined as interval data. Sullivan and Artino [33] support examining survey data in this way and conclude that “parametric tests can be used to analyze Likert scale response.” This conclusion is also supported by others in the medical education and assessment and evaluation literature [34, 35]. Notwithstanding, care should always be taken when examining self-reported survey data. Finally, this study incorporated the use of multiple statistical tests. The Bonferroni correction was applied to help account for the increased likelihood of Type I errors [23, 24]. Still, future studies may benefit from using a more streamlined survey instrument which would help to manage the number of statistical comparisons. Although these limitations need to be considered, the study was based on founded assessment approaches, had a relatively large sample size, and met all necessary assumptions required for statistical analysis.

### Limitations of design and delivery of student-led curriculum

Finally, it is important to address limitations with the design and delivery of student-led curriculum. Fletcher et al. explain that one of the main drawbacks to a student-led curriculum is that students lack the clinical knowledge and professional experience to fully develop the curriculum [9]. Without this background, there may be gaps within the curriculum that require oversight and review from faculty and staff. Taking this into account, we made sure all curricular materials were reviewed by faculty experts and all synchronous sessions were led by clinicians and faculty in the Fundamentals of COVID-19 course. Another limitation to student-led curriculum is the high turnover rate in student participation. Student-led curricular efforts require considerable resources including a commitment of faculty, staff, and other relevant stakeholders to longitudinal mentorship. Although high student turnover was not as limiting for our project as others, the COVID-19 pandemic did necessitate a shortened project timeline. This may have resulted in students not having longitudinal mentorship experiences that were appropriate for the scale of the project. Future student-led curricular projects should be mindful of these potential limitations and include opportunities for faculty feedback and guidance.

## Conclusions

COVID-19 presented medical educators with the barrier of extended lapses of time in in-person student learning. This new reality occurred at a time when faculty were also being met with increased clinical duties due to a global pandemic. In this time of change, students were able to provide critical leadership by creating a highly effective virtual Fundamentals of COVID-19 course. The student-led course served medical students [36] by facilitating acquisition of the knowledge, skills, and abilities, necessary to respond to the pandemic. The Fundamentals of COVID-19 course provided a quintessential example of the ability of students and faculty to work together to fill an educational gap caused by COVID-19. Future research and curricular development should continue to examine student-led approaches and virtual instruction in medical education. A sustained commitment to these goals ensures ever improving training techniques for future physicians through innovative and evidence-based curriculum.

## Supplementary Information

**Additional file 1.** Fundamentals of COVID-19 Modules Mapped to Course Learning Objectives, Institutional Indiana University School of Medicine Competencies. Table providing the course learning objectives of IUSM’s Fundamentals of COVID-19 course, along with the IUSM Institutional Learning Objective to which the CLO maps, and the course module(s) addressing that CLO.

**Additional file 2.** Pre- and Post- Survey Questions. Pre-course and post-course survey tools used to gather data for this study.

## Data Availability

The datasets used and/or analyzed during the current study are available from the corresponding author on reasonable request.

## Abbreviations

IUSM: Indiana University School of Medicine
KSA: Knowledge, Skills, and Abilities
CLO: Course Learning Objective
SCRT: Student Curriculum Review Team
GPA: Grade Point Average
IV: Independent Variable
DV: Dependent Variable
N: Study Size
M: Mean
SD: Standard Deviation
CI: Confidence Interval
OSCE: Objective Structured Clinical Exams
PBA: Performance Based Assessments
CFMS: Canadian Federation of Medical Students
