# Supplementary material for: Student-led curricular approaches in medical education: the educational effects of a virtual fundamentals of COVID-19 course
Source: BMC Med Educ. 2022 Mar 8;22:158. doi: 10.1186/s12909-021-03076-x (PMC8902280; doi:10.1186/s12909-021-03076-x)
Supplement: Supplementary file 1 — Additional file 1. Fundamentals of COVID-19 Modules Mapped to Course Learning Objectives, Institutional Indiana University School of Medicine Competencies. Table providing the course learning objectives of IUSM’s Fundamentals of COVID-19 course, along with the IUSM Institutional Learning Objective to which the CLO maps, and the course module(s) addressing that CLO. [file 12909_2021_3076_MOESM1_ESM.pdf]

Additional File 1. Fundamentals of COVID-19 Modules Mapped to Course Learning Objectives, Institutional Indiana University School of Medicine Competencies.

Institutional Competency	Course Learning Objective	Associated Learning Modules
**Medical Knowledge (MK)**	1) Explain the principles of virology and immunology as they relate to COVID-19.	Virology and Immunology
	2) Identify the causal agents and the management of epidemics and pandemics, including the process of vaccine development in modern medicine.	History of Epidemics and Pandemics in Modern Medicine
		Principles of Disaster Management
		Laboratory Science
	3) Explain the clinical presentation and the pathophysiology of the COVID-19 pandemic.	Virology and Immunology
		Patient Care/Radiology
		Laboratory Science
	4) Evaluate treatment and disease management options using principles of Evidence-Based Medicine (EBM) to apply the latest data for suspected and confirmed COVID-19 patients.	Patient Care/Radiology
		Evidence-Based Medicine Review of Literature on Disease
	5) Use principles of evidence-based medicine, including biostatistics, to evaluate the efficacy and potential for therapeutic and diagnostic interventions for COVID-19 infection.	Virology and Immunology
		Patient Care/Radiology
		Specialty Considerations during the COVID-19 Pandemic
		Evidence-Based Medicine Review of Literature on Disease
		Laboratory Science
	6) Apply the science of epidemiology and analyze the management of epidemics and pandemics historically and in modern medicine.	Epidemiology of Disease
		History of Epidemics and Pandemics in Modern Medicine
		Principles of Disaster Management
**Patient Care (PC)**	7) Analyze clinical presentation and pathologic findings, determine the disease entity, outline a treatment course for COVID19 patients.	Patient Care/Radiology
	8) Recognize common laboratory, radiologic and clinical presentations of disease of COVID-19 patients.	Patient Care/Radiology
		Laboratory Science
	9) Identify at-risk populations for poor outcomes with COVID-19 and preventative measures for these people.	Epidemiology of Disease
		Specialty Considerations during the COVID-19 Pandemic

**Practice-Based Learning and Improvement (PBLI)**	10) Engage in self-directed learning by identifying a research question, appraising the quality and credibility of sources to answer the research question, and synthesizing the relevant information to advance the understanding of pandemic responses.	Epidemiology of Disease
**Interpersonal and Communication Skills (ICS)**	11) Identify ways of modifying strategies for communicating about an evolving healthcare topic based on the context and audience.	Telehealth
		Communication Considerations during COVID-19
	12) Share evolving information about the COVID-19 pandemic accurately through appropriate media.	Telehealth
		Communication Considerations during COVID-19
**Systems-Based Practice (SBP)**	13) Outline the roles of medical professionals and non-medical professionals in responding during epidemics/pandemics.	Principles of Disaster Management
		Laboratory Science
		Ethical Considerations during a Pandemic
		Public Policy during the COVID Pandemic
	14) Evaluate the impact of population health and social determinants of health in the context of a pandemic, and in particular for COVID-19.	Epidemiology of Disease
		Patient Care/Radiology
		Specialty Considerations during the COVID-19 Pandemic
	15) Identify ways in which individuals and organizations can advocate at the state and national level during epidemics/pandemics.	Principles of Disaster Management
		Ethical Considerations during a Pandemic
		Public Policy during the COVID Pandemic
	16) Describe disaster medicine principles, including the processes and policies by which community and international agencies interact to coordinate safe and effective disaster/pandemic response.	Specialty Considerations during the COVID-19 Pandemic
		Principles of Disaster Management
		Appropriate Personal Protective Equipment Protocol
		Public Policy during the COVID Pandemic
	17) Describe the utilization and preservation of finite resources during disaster/pandemic responses.	Specialty Considerations during the COVID-19 Pandemic
		Principles of Disaster Management
		Appropriate Personal Protective Equipment Protocol
		Ethical Considerations during a Pandemic
		Public Policy during the COVID Pandemic

**Professionalism (P)**	18) Outline the traits and behaviors of leaders within the medical field.	Leadership and Teamwork Traits during a Pandemic
	19) Identify basic strategies for mental health and wellbeing promotion for providers in the face of a healthcare emergency and understand their importance to overall health.	Wellness and Self-Care for Healthcare Providers during a Pandemic
	20) Debate the legal, psychosocial, and ethical aspects that impact COVID-19 patients, providers, and the community during the pandemic, in particular the issues surrounding resource utilization.	Specialty Considerations during the COVID-19 Pandemic
		Principles of Disaster Management
		Ethical Considerations during a Pandemic
		Public Policy during the COVID Pandemic

4
