# Supplementary material for: Student-led curricular approaches in medical education: the educational effects of a virtual fundamentals of COVID-19 course
Source: BMC Med Educ. 2022 Mar 8;22:158. doi: 10.1186/s12909-021-03076-x (PMC8902280; doi:10.1186/s12909-021-03076-x)
Supplement: Supplementary file 2 — Additional file 2. Pre- and Post- Survey Questions. Pre-course and post-course survey tools used to gather data for this study. [file 12909_2021_3076_MOESM2_ESM.pdf]

Additional File 2: Pre- and Post- Survey Questions

**Pre- and Post- Survey KSA Questions:**

Knowledge, Skills, and Abilities as they relate to COVID-19

Please indicate your level of agreement with the following statements regarding your current knowledge, skills, and abilities as they relate to foundational concepts in this course. These questions are directly tied to the Course Learning Objectives (CLOs).

1= Strongly Disagree; 2= Disagree; 3= Neutral; 4=Agree; 5=Strongly Agree

*Knowledge*

1. I am knowledgeable about virology and immunology as they relate to COVID-19.
2. I am knowledgeable about the pathophysiology of COVID-19.
3. I am knowledgeable about the impact of population health in the context of a pandemic, and in particular for COVID-19.
4. I am knowledgeable about the impact of social determinants of health in the context of a pandemic, and in particular for COVID-19.
5. I am knowledgeable about the ways individuals and organizations can advocate at the state and national level during epidemics/pandemics.
6. I am knowledgeable about the legal aspects that impact COVID-19 patients, providers, and the community during the pandemic.
7. I am knowledgeable about the issues surrounding utilization and preservation of finite resources that impact patients, providers, and the community during a pandemic.

*Skills*

8. I am confident in my skills to use principles of evidence-based medicine, including biostatistics, to evaluate efficacy of therapeutic interventions for COVID-19 infection.

9. I am confident in my skills to analyze the management of epidemics and pandemics historically and in modern medicine.
10. I am confident in my skills to identify a research question.
11. I am confident in my skills to appraise the quality and credibility of a source, and synthesize the information to advance my understanding of pandemic responses.
12. I am confident in my skills to implement basic strategies for mental health and wellbeing promotion for providers in the face of a healthcare emergency, and understand their importance to overall health.

*Abilities*

13. I am confident in my ability to recognize the clinical presentation of a patient with COVID-19.
14. I am confident in my ability to outline a treatment course for suspected COVID-19 patients.
15. I am confident in my ability to identify at-risk populations for poor outcomes with COVID-19.
16. I am confident in my ability to describe disaster medicine principles, including the processes and policies by which community and international agencies interact to coordinate a safe and effective disaster/pandemic response.
17. I am confident in my ability to identify ways of modifying communication strategies based on the context.

**Additional Post-Course Survey Questions:**

*Meaningful Session*

1. The (Blank) session or course module was the most meaningful because I learned (Blank).

*Teamwork and Group Interactions*

2. Please rate your experience working on a team with medical students of a different year.

(Scale: Extremely unfavorable, unfavorable, favorable, very favorable, extremely favorable)

3. Please rate your team's ability to work together.

(Scale: Very poor, poor, average, strong, very strong)

*Short Answer Questions*

4. Please describe the benefits of working on a team with medical students of a different year.
5. Please describe the disadvantages of working on a team with medical students of a different year.

*Virtual Delivery of Course Content*

(Scale: Strongly Disagree; Disagree; Neutral; Agree; Strongly Agree)

6. The technology generally worked well for this virtual course.
7. The content was delivered effectively in a virtual format.

*Overall Course Effectiveness*

(Scale: Strongly Disagree; Disagree; Neutral; Agree; Strongly Agree)

8. Overall, this course provided an effective learning experience.
9. The virtual delivery of course content did not detract from the overall effectiveness of this class.

*Written Feedback*

10. Please describe what worked well in this course. Provide specific examples where possible.
11. Please describe how you will apply what you learned in this class to your future medical practice. Provide specific examples where possible.
12. Please provide suggestions for improving this course. Provide specific examples where possible.
